# The EU global health strategy: from policy to implementation

**DOI:** 10.1186/s41256-025-00410-4

**Published:** 2025-02-25

**Authors:** Jolene Skordis, Guenter Froeschl, Sante Leandro Baldi, Astrid Berner-Rodoreda, Nuria Casamitjana, Frank Cobelens, Kerstin Klipstein-Grobusch, Mario Raviglione, Alberto Rocamora, Anne-Mieke Vandamme, Antoni Plasència

**Affiliations:** 1 Institute for Global Health, University College London, London, UK; 2 Institute of Infectious Diseases and Tropical Medicine, University Hospital, Ludwig-Maximilians-Universität, Munich, Germany; 3 Centre for Multidisciplinary Research in Health Science, University of Milan, Milan, Italy; 4 Heidelberg Institute of Global Health, Heidelberg University, Heidelberg, Germany; 5 Barcelona Institute for Global Health, ISGlobal, Hospital Clínic - Universitat de Barcelona, Barcelona, Spain; 6 Amsterdam Institute for Global Health and Development, Amsterdam, The Netherlands; 7 Julius Global Health, Department of Global Public Health and Bioethics, Julius Center for Health Sciences and Primary Care, University Medical Center Utrecht, Utrecht University, Utrecht, The Netherlands; 8 Clinical and Epidemiological Virology, Institute for the Future, Rega Institute for Medical Research, KU Leuven, Leuven, Belgium

**Keywords:** European union, Global health, Strategy, Policy

## Abstract

In November 2022, a draft of the next EU Global Health Strategy was published. The European Global Health Research Institutes Network (EGHRIN) of 22 leading European Universities has analysed the Strategy. In this commentary, EGHRIN notes the Strategy’s positive commitments to life-course wellbeing, strengthening health systems and combating health threats in an equitable and collaborative manner. We find the strategy is compatible with the sustainable development goals and addresses social, political and environmental determinants of health. However, our analyses also highlight a lack of critical assessment of the private sector’s role within health systems, insufficient attention to syndemics, and little emphasis on transdisciplinary education and the ethical training of a global health workforce. We conclude that, while its commitments are commendable, the greatest challenge for the next EU Strategy will be in its implementation. The trategy currently lacks a fully-resourced and clearly hypothecated funding mechanism and there is little evidence to date of the stated commitment that Global Health will be considered in all EU policy areas. In the present geopolitical climate, a speedy acceptance of both the policy and an implementation plan is needed more than ever.

## Background

Horizon 2020 was the EU's Framework Programme for Research and Innovation, which ran from 2014 to 2020, with a budget of nearly EUR 80 billion. Horizon Europe is the successor to Horizon 2020 and the EU’s current key funding programme for research and innovation. The European Commission reports that funding for Horizon Europe over the period 2021–2027, is EUR 93.5 billion. Both Horizon 2020 and Horizon Europe have had the potential to shape the focus of innovation and knowledge creation. In the context of health, both programmes have represented significant commitments to Global Health research and innovation with budgets of around €4 billion and €8.3 billion for health respectively [1].

Launching the next EU Global Health Strategy as part of the EU Global Gateway, reflects the broader consideration that the European Commission gives to health [2]. The previous EU policy dates from 2010, with significant global changes in the interim. The content of the new strategy suggests a “change in mind-set” with the urgent need to reframe health in a comprehensive manner with commitment to sustainable institutional change. The new strategy has three main priorities: (I) deliver better health and well-being of people across their life course; (II) strengthen health systems and advance universal health coverage; (III) prevent and combat health threats, including pandemics. In addition, 20 guiding principles are outlined, including collaboration with low- and middle-income countries (LMICs), a transdisciplinary approach to innovation and a One Health perspective [3]. Additionally, the Commission regards the proposed Pandemic Agreement by the World Health Organization (WHO) as a crucial element in attaining fair and equitable access globally to healthcare products [4].

Following the official launch of the next EU Global Health Strategy, the European Global Health Research Institutes Network (EGHRIN), an association of 22 European higher education institutions engaged in Global Health research, reflects on the Strategy's priorities in a complex geopolitical landscape. The reflection also offers a commentary on the Strategy’s strengths and expected challenges [5].

## Critical assessment

According to the High Representative of the Union for Foreign Affairs and Security Policy, Global Health needs to be reshaped as a geopolitical issue: “*Health has become a critical element of foreign, security and trade policies*.”[6] However, we would like to critically recall the securitisation of health that followed the 9/11 attacks in the United States and the “Macroeconomics and Health” agenda that shaped health investment in the first decade of the 21st Century. The former promoted health investment only as a means to achieving national security, while the latter promoted health investment primarily as a means to increasing national wealth. Neither acknowledged the human right to health, and both demonstrated how Global Health is vulnerable to being co-opted by moving agendas. We urge a consistent focus on global health risks and emergencies, which require longer term commitment to build trusting partnerships and healthy futures, not only within the European Union, but also globally. EGHRIN urges stakeholders to place greater emphasis on research & innovation, and education & training to address the complex physical, mental, social, commercial, legal and political determinants of health, a need that became evident during the COVID-19 pandemic [7].

The EU Global Health Strategy suggests a shift in paradigm with a need to view health policies in a more comprehensive and holistic manner. This is an opportunity to deploy much needed synergies between universal health coverage, health security and health promotion. Nevertheless, the efforts to provide global access to COVID-19 vaccines have taught us that the establishment of multilateral instruments alone is insufficient [8]. The economic and security risks in many low-income countries which the EU would aim to support, will challenge efforts at health systems strengthening. The litmus test will be the new EU’s global collaboration and solidarity principles based on equity rather than a “member-states first” policy.

While the Strategy addresses the prevention and treatment of communicable and non-communicable diseases, a particular issue for many LMICs are the “syndemics” of communicable and non-communicable diseases, and their intersection with the economic, social and environmental causes of ill-health and disease. We endorse the Strategy’s acknowledgement that Global Health will be increasingly impacted by climate change and that a human rights-based approach needs to be upheld in addressing these impacts. Recent debate within the EU about migrants and asylum seekers may challenge this approach and again, there is a current risk that the positive aims of the strategy are either substantively diluted or lost in practice. We encourage the consideration of funding mechanisms and other practical levers to ensure that the holistic perspective describe previously, is not lost on the journey from policy to implementation.

The European Global Health Research Institutes Network also welcome plans to strengthen the use of digital health in LMICs to improve national and global responses. However, given existing challenges such as internet connection, electricity supplies and data protection in a number of countries, access to offline data and sustainable solutions (e.g., solar energy) need to go hand-in-hand with the expansion of digital technology. The Strategy does not currently assess the risks of collaboration with the private sector, either for the delivery of digital health or for health service provision more widely, which requires further study before the opportunities can be fully maximised and the risks avoided.

In the current strategy, EU Member States are responsible for financing. As the available funds for health in the current Multiannual Financial Framework have been largely exhausted, the success of subsequent fund-raising rounds will be of critical importance [9]. Furthermore, the strategy has been launched during a period of deep ideological divisions within the European Union. The first attempted Council conclusions drafted by the Swedish Presidency amounted to an endorsement of the European Commission proposal. While the document satisfied most Member States, there was a deadlock given the opposition by one due to explicit references to sexual and reproductive health [10]. Eventually, the Belgian Presidency managed to secure the endorsement of all Member States [11]. This process shows that Global Health policies are sensitive to political change and at risk of being deprioritised, or fragmented. A strengthening of populist groupings in the recent 2024 European election and a new composition of the European Commission will test the resilience of the Strategy.

## Policy and implementation challenges

The EU Global Health Strategy offers numerous important opportunities for progress in Global Health. However, to be fully effective it should also address the following five policy and implementation challenges:

(I) *Global Health in all policies*: The Strategy states that the “*Commission will fully integrate global health considerations into all EU policy areas*” [2]. How will this intention be reconciled with the EU’s free trade agreements, that impose extended patent terms and delay the market entry of generic medication that is usually corresponding to lower cost [12]? Even the Commission’s proposed Pharma Reform of 2023 included little consideration of the global health strategy and its principles, until it was amended by the European Parliament [13]. It will be an ongoing challenge to ensure that Global Health is indeed considered by policy groupings that may have previously considered themselves separate from Global Health priorities.
(II) *Reframe Global Health as a geopolitical issue*: While the Strategy acknowledges a changing geopolitical environment (Publ. Office of the EU 2022), recent EU and US elections pose new challenges. With US funding for multilateral organisations becoming increasingly uncertain, the envisaged global coordinating role of the World Health Organisation will rely more heavily on EU funding and on galvanizing support from other countries and regions. This too will be challenging as differing political agendas within the EU, make it more unlikely that “Member States [are able] to speak with one voice” (Publ. office of the EU, 2022). Migration, health securitization, private sector access to global health markets, misinformation and disagreement about climate risks to health will all serve to polarise political support for global health action. For the WHO to set its agenda independently, public funding is essential and political consensus needs to be built around the strategy and its implementation.
(III) *Broaden the focus to Planetary Health*: Syndemics are becoming an increasing challenge for many low- and middle-income countries but are not highlighted in the Strategy. While the Strategy emphasizes zoonoses and environmental risks and the need for collaboration, it needs to go further in promoting inter- and multi-disciplinary solutions. Successfully addressing infectious agents alongside non-communicable diseases, will necessitate collaboration across academic and policy sectors with regard to human and animal health, and environmental surveillance and protection.
(IV) *Emphasise pandemic preparedness through global cooperation and a societal approach*: The COVID-19 pandemic has given the global community a good example of shortcomings in global cooperation and a society-wide approach when addressing a Global Health issue. The EU Global Health Strategy rightly stresses the cross-border nature of a pandemic and supports equitable access to and stockpiling of resources to bring a pandemic under control. However, we argue that the current emphasis on global health security is insufficiently taking into account the need for a society-wide preparedness through co-creation of measures, empathic communication and improving reliable information sharing. Strengthening health systems resilience includes building trust, which must be addressed world-wide and society-wide.
(V) *Invest in local transdisciplinary education and training*: In previous health emergencies, such as the Ebola Virus Disease outbreak in 2014–2016 or the COVID-19 Pandemic, common response limitations included a shortage of local health workers, the lack of a local academic landscape to support surveillance and knowledge generation, and local communities with little health literacy and agency. Given that the strategy promotes “*mutually beneficial mobility arrangements*” [2] in the context of an ageing population in Central and Northern Europe, skills shortages in the global south are likely to be amplified. This needs to be acknowledged, and both local training and ethical recruitment actively supported.

## Conclusions

During times of scarcity and with an increasingly polarised political landscape, efforts should be made to guarantee resources for the implementation of the EU Global Health Strategy. Provision for supporting activities should be made in EU funding instruments, including the upcoming Framework Programme 10, starting in 2028. Only with continued political engagement and support can its implementation be successful, promoting innovation and global cooperation to improve the future of all people and the planet on which we depend.

## Data Availability

Not applicable.

## Abbreviations

EGHRIN: European global health research institutes network
EU: European union
LMIC: Low- and middle-income country
WHO: World health organization
